# Facile Synthesis of MnO_2_ Nanoflowers/N-Doped Reduced Graphene Oxide Composite and Its Application for Simultaneous Determination of Dopamine and Uric Acid

**DOI:** 10.3390/nano9060847

**Published:** 2019-06-02

**Authors:** Xuan Wan, Shihui Yang, Zhaotian Cai, Quanguo He, Yabing Ye, Yonghui Xia, Guangli Li, Jun Liu

**Affiliations:** 1 College of Life Sciences and Chemistry, Hunan University of Technology, Zhuzhou 412007, China; wanxuan1111@163.com (X.W.); yangshihui0522@163.com (S.Y.); caizhaotian1998@163.com (Z.C.); hequanguo@126.com (Q.H.); yyb980501@163.com (Y.Y.); 2 Zhuzhou Institute for Food and Drug Control, Zhuzhou 412000, China; Sunnyxia0710@163.com

**Keywords:** dopamine, uric acid, MnO_2_ nanoflowers, N-doped reduced graphene oxide, voltammetric sensor

## Abstract

This study reports facile synthesis of MnO_2_ nanoflowers/N-doped reduced graphene oxide (MnO_2_NFs/NrGO) composite and its application on the simultaneous determination of dopamine (DA) and uric acid (UA). The microstructures, morphologies, and electrochemical performances of MnO_2_NFs/NrGO were studied using X-ray diffraction (XRD), scanning electron microscopy (SEM), cyclic voltammetry (CV), and electrochemical impedance spectroscopy (EIS), respectively. The electrochemical experiments showed that the MnO_2_NFs/NrGO composites have the largest effective electroactive area and lowest charge transfer resistance. MnO_2_NFs/NrGO nanocomposites displayed superior catalytic capacity toward the electro-oxidation of DA and UA due to the synergistic effect from MnO_2_NFs and NrGO. The anodic peak currents of DA and UA increase linearly with their concentrations varying from 0.2 μM to 6.0 μM. However, the anodic peak currents of DA and UA are highly correlated to the Napierian logarithm of their concentrations ranging from 6.0 μM to 100 μM. The detection limits are 0.036 μM and 0.029 μM for DA and UA, respectively. Furthermore, the DA and UA levels of human serum samples were accurately detected by the proposed sensor. Combining with prominent advantages such as facile preparation, good sensitivity, and high selectivity, the proposed MnO_2_NFs/NrGO nanocomposites have become the most promising candidates for the simultaneous determination of DA and UA from various actual samples.

## 1. Introduction

Dopamine (DA) and uric acid (UA) often coexist in the biological fluids, such as blood serum, urine, and extracellular fluids, which play a vitally significant role on the regulation of human physiological functions and metabolic activities [1]. As an essential catecholamine neurotransmitter, DA plays a pivotal role in regulating the functions of cardiovascular and central nervous systems, adjusting emotions, and maintaining hormonal balances [2]. The dysfunction of DA possibly causes many neurological disorders like Parkinson’s syndrome, Alzheimer’s diseases, and schizophrenia [3,4,5]. For a heathy individual, the DA levels in biological matrixes generally vary from 0.01 μM to 1 μM. The response signals of DA are often susceptible to interferences from endogenous biomolecules i.e., ascorbic acid (AA) and UA. Therefore, it remains a great challenge for the fast and precise detection of DA. As another critical biomolecule in human body, UA is commonly regarded as the metabolic product of purine [6]. Generally, the UA level is 4.1 ± 8.8 mg/100 mL for a healthy individual [7]. The abnormal concentration of UA in physiological fluids likely leads to several disorders including pneumonia, hyperuricemia, and gout [8]. Thus, the levels of DA and UA in physiological fluids have become important indicators or biomarkers for healthcare and clinical diagnosis. Therefore, it is extremely necessary to propose some efficient and reliable approaches toward the simultaneous determination of DA and UA. 

Up until now, various detection approaches have been reported for detecting DA and UA, such as chemiluminescent [9], HPLC [10,11], fluorometry [12], spectrophotometry [13], and surface plasmon resonance [14]. These techniques are very reliable, but they often involve cumbersome and time-consuming procedures that require large instruments, experienced technicians, and even a large amount of poisonous solvents [15]. Recently, electrochemical approaches have drawn growing attention for the determination of bioactive compounds, food dyes, and pollutants, owing to their considerable advantages such as being inexpensive, facial operation, high efficiency, good selectivity, and sensitivity [16,17,18,19,20]. In addition, DA and UA are highly electroactive biomolecules, which are more suitable for electrochemical detection. However, bare electrodes often suffer from electrode fouling and cross-interference issues, which result in poor sensitivity and reproducibility [7,21]. To address the issues, various nanomaterials were developed to construct electrochemical sensors. 

As a versatile transition metal oxide, MnO_2_ has been intensively utilized in energy storage, catalysis, and sensors because of its peculiar properties including low-cost, more abundance, high-catalytic activity, and environmental friendliness. Until now, a variety of nanostructured MnO_2_ such as nanowires [16,22], nanorods [17,23,24], nanotubes [25,26], microspheres [27,28], and nanoflowers [29,30] have been prepared, characterized, and even used in electrochemical determination. Among these morphologies, MnO_2_ nanoflowers (MnO_2_NFs) have drawn considerable attention, attributing to their pore structure and large specific surface area. As sensing materials, MnO_2_ nanoflowers have been used for the detection of lead ion [29], ractopamine [30], salbutamol [30], guaiacol [31], vanillin [31], hydrogen peroxide [32], and DA [33]. These studies demonstrate that MnO_2_ nanoflowers improve the electrochemical performances significantly. But their poor dispersibility and electrical conductivity have impeded widespread applications in electrochemical sensors. 

To resolve this problem, an effective strategy is to composite nanostructured MnO_2_ with graphene materials, which not only effectively improve the dispersibility, but also endow a synergistic effect towards sensing target analytes. However, the electrical conductivity of graphene cannot be fully controlled due to the lack of bandgap [34]. In this regard, many approaches have been proposed to modify the electron transfer and surface chemical properties, among which the doping of nitrogen into graphene has displayed enormous potential for widespread applications [35]. Compared to pristine graphene, N-doped reduced graphene oxide (NrGO) possesses a more biocompatible C-N microenvironment, a much larger functional surface area, a better electrical conductivity, a higher ratio of surface-active groups to volume, and enhanced electrocatalytic effects [35,36]. Therefore, NrGO has been widely used to construct a variety of electrochemical sensors. For example, Yang and coworkers [37] reported a facie one-step hydrothermal preparation of Fe_2_O_3_/NrGO nanohybrids toward DA detection. Fe_2_O_3_/NrGO showed superior electrocatalytic activity toward DA oxidation, with a broad detection range (0.5 μM–0.34 mM), a low limit of detection (LOD, 0.49 μM), and good sensitivity (418.6 μA mM^−1^ cm^−2^). Chen et al. [38] prepared NrGO/MnO nanocomposite via the freeze-drying technique to construct a selective electrochemical sensor for the detection of DA in the coexistence of UA and AA. Although NrGO has been intensively utilized in electrochemical sensing, as far as we know there is no report available for the use of MnO_2_/N-doped graphene composite for the simultaneous detection of DA and UA.

Herein, MnO_2_NFs/NrGO nanocomposites were prepared by a facile, cost-effective and highly efficient route rather than the conventional hydrothermal method. Specifically, MnO_2_NFs were prepared by a slow addition of MnSO_4_ into KMnO_4_ solution followed by a simple stirring procedure, then composited with NrGO nanosheets with an ultrasonication assistant. The combined virtues of MnO_2_NFs and NrGO nanosheets are expected to enhance electrochemical sensing properties, which has been proven by using the MnO_2_NFs/NrGO as an efficient electrocatalyst for the simultaneous determination of DA and UA in serum samples. The proposed sensor showed remarkable catalytic capacity toward the oxidation of DA and UA, with two detection ranges (0.2–6.00 μM and 6–100 μM), low LOD (36 and 29 nM for DA and UA respectively), and good selectivity as well as reproducibility.

## 2. Materials and Methods

### 2.1. Reagents

UA, DA, NaH_2_PO_4_, and Na_2_HPO_4_ were purchased from Aladdin Reagents Co., Ltd. (Shanghai, China). K_4_[Fe(CN)_6_], K_3_[Fe(CN)_6_], MnSO_4_, KMnO_4_, NaOH, H_3_PO_4_, and absolute ethanol were supplied by Sinopharm Chemical Reagent Co. Ltd. (Shanghai, China). All of the chemicals were analytically pure and used as received. NrGO was supplied by Nanjing Xianfeng NANO Material Tech Co. Ltd. (Nanjing, China). Human serum samples were provided by Zhuzhou People’s Hospital (Zhuzhou, China). The human samples are a mixture of residual serum from various individuals after clinical examination. Deionized water with the resistivity of 18.2 MΩ was used in all of the experiments.

### 2.2. Materials Characterization

Crystalline structures and surface morphologies of MnO_2_NFs and MnO_2_NFs/NrGO were investigated by powder X-ray diffractometry (XRD) and scanning electron microscopy (SEM), respectively. SEM images were taken from a cold field-emission SEM (Hitachi S-4800, Tokyo, Japan). The XRD patterns of MnO_2_NFs were collected using a powder XRD system (PANalytical, Almelo, The Netherlands) with monochromatized Cu Kα radiation (λ = 0.1542 nm), which was operated at 40 kV and 40 mA. 

### 2.3. Synthsis of MnO_2_NFs/NNrGO Comoposites

The MnO_2_NFs was prepared by a slow addition of MnSO_4_ into KMnO_4_ solution followed by a simple stirring procedure. Typically, 1 mmol of KMnO_4_ and 1.5 mmol of MnSO_4_ were adequately dissolved into 20 mL deionized water, separately. Then, the MnSO_4_ solution was added dropwise into KMnO_4_ solution at a rate of 1 mL min^−1^, and agitated continuously at room temperature for 2 h. The resultant product was collected by centrifugation at 12,000 rpm, followed by cleaning alternately with absolute alcohol and deionized water three times, and dried at 60 °C in a vacuum oven overnight. Obviously, this route is time-saving and more convenient when compared with the conventional hydrothermal method. 

MnO_2_NFs/NrGO composites were prepared as follows. Firstly, 10 mg MnO_2_NFs were uniformly dispersed in 10 mL deionized water under an ultrasonication bath for 0.5 h. Then 0.2 g NrGO nanosheets were added into the above MnO_2_NFs dispersion (1 mg mL^−1^) and dispersed under ultrasonication for 1 h. The MnO_2_NFs/NrGO were stored at 4 °C in a refrigerator when not used. To ensure good reproducibility, the MnO_2_NFs/NrGO were subjected to ultrasonication for 0.5 h before each modification.

### 2.4. Fabrication of MnO_2_NFs/NrGO Modified Electrodes

The bare glassy carbon electrodes (GCEs) were carefully polished using 0.3 μm and 0.05 μm alumina slurry, then alternately washed by anhydrous alcohol and deionized water several times, and allowed to dry under an infrared lamp. The MnO_2_NFs/NrGO-modified GCE (MnO_2_NFs/NrGO/GCE) was prepared via a simple drop-casting approach. Specifically, 5 μL MnO_2_NFs/NrGO dispersion was carefully dropped and casted on the GCE surface with a micropipette, then dried with an infrared lamp to form a sensing film. For comparison, MnO_2_NFs and NrGO-modified GCEs (MnO_2_NFs/GCE, NrGO/GCE) were also prepared via similar procedures.

### 2.5. Procedures for Electrochemical Mesurements

For all the electrochemical tests, a typical three-electrode assemble was immersed into a 10 mL electrochemical cell, in which a bare or modified GCE was worked as the working electrode. Saturated calomel electrode (SCE) and platinum wire were used as a reference electrode and auxiliary electrode, respectively. To evaluate the electrochemical performance of various modified electrodes, cyclic voltammetry (CV) and electrochemical impedance spectroscopy (EIS) was measured in the 0.1 M phosphate buffered solution (PBS, pH 7.0), using 0.5 mM [Fe(CN)_6_]^3−/4−^ as redox probe couples. EIS plots for different electrodes were recorded at open circuit potential using 5 mV (rms) AC sinusoid signal at a frequency range from 100,000 Hz to 0.1 Hz. The voltammetric responses of 10 μM DA and UA at different electrodes were tested by CV. After a suitable accumulation, linear scanning voltammetry (LSV) was performed for the determination of DA and UA. The potentials were scanned from 0 V to 0.8 V at 100 mVs^−1^ for both CV and LSV. 

## 3. Results and Discussion

### 3.1. Physical Chararazation

**Figure 1:**
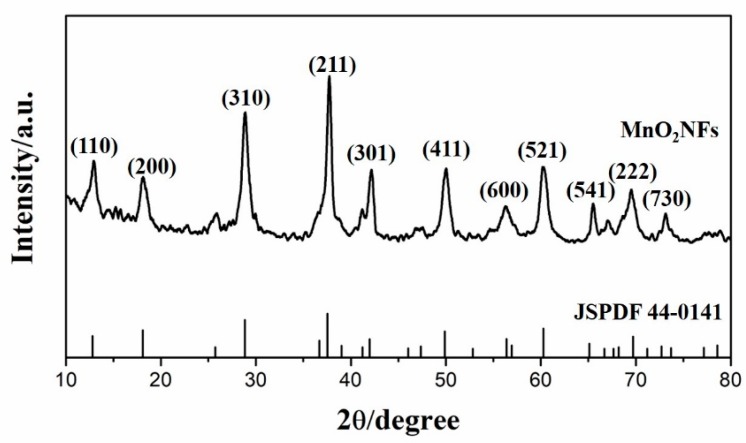
X-ray diffraction (XRD) patterns of MnO_2_ nanoflowers and standard card JSPDF 44-0141.

**Figure 2:**
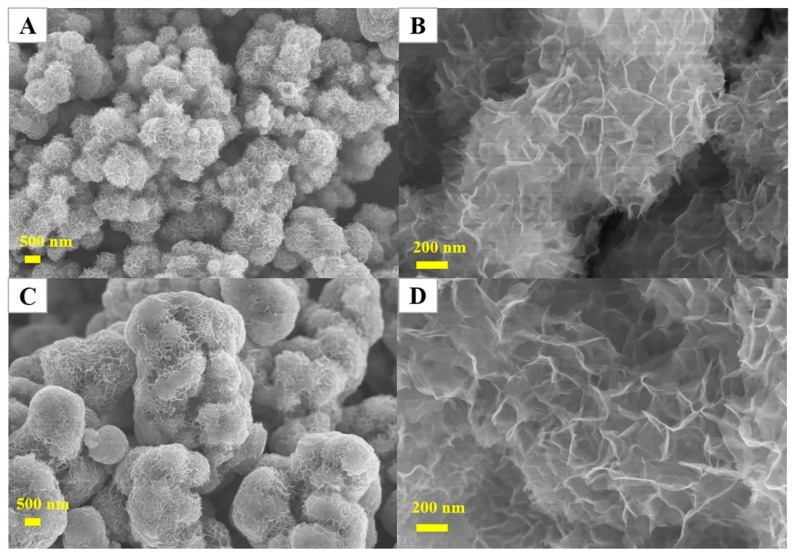
Scanning electron microscopy (SEM) images of MnO_2_NFs (**A**,**B**) and MnO_2_NFs/NrGO nanocomposites (**C**,**D**) at different magnifications.

The crystalline structure of MnO_2_ nanoflowers was characterized by XRD. As presented in Figure 1, sharp diffraction peaks were observed at 2θ of 12.94°, 18.34°, 28.78°, 37.66°, 42.14°, 49.90°, 56.44°, 60.26°, 69.74°, 71.34°, and 73.72°, which can be well-indexed into (110), (200), (310), (211), (301), (411), (600), (521), (541), (222), and (730) facets, respectively. It is in good agreement with XRD standard card JSPDF 44-0141 [16,17], suggesting tetragonal crystalline of α-MnO_2_ were successfully synthesized. Moreover, no visible peak relating to impurities appears, indicating high-purity of α-MnO_2_. SEM images of MnO_2_NFs are shown in Figure 2A, B. Obviously, flower-like nanostructures composed of interconnected nanoflakes suggests MnO_2_ nanoflowers were successfully synthesized. The porous microstructures indicate that MnO_2_ nanoflowers have a large specific surface area, which is favorable for electrochemical sensing. As shown in Figure 2C,D, the NrGO nanosheets were warped on the surface of MnO_2_NFs, suggesting MnO_2_NFs/NrGO nanocomposites were successfully prepared. 

### 3.2. Evaluation of Electrochemical Performances

**Figure 3:**
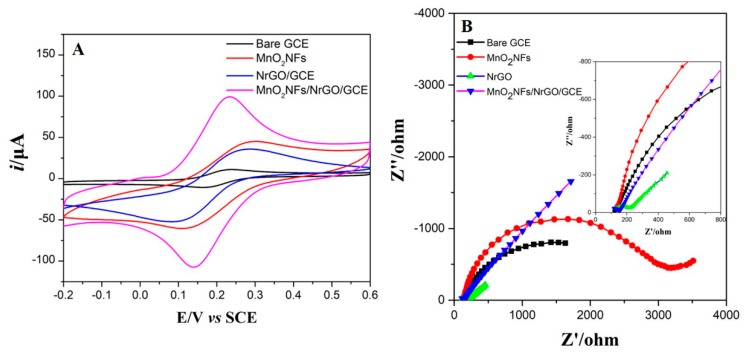
Cyclic voltammetry (CVs) (**A**) and Nyquist plots (**B**) for various electrodes in 0.5 mM [Fe(CN)_6_]^3−/4−^ containing 0.1 M KCl solution. The inset in Figure 3B represents the magnification of Nyquist at a higher frequency.

In order to assess the electrochemical performances, CVs for various modified electrodes were measured in a mixture solution of 0.5 mM [Fe(CN)_6_]^3−/4−^ and 0.1 M KCl (Figure 3A). A pair of quasi-reversible redox peaks occurred on all of the electrodes with *i*_pa_/*i*_pc_ ≈ 1.0. At bare GCE, a pair of weak redox appeared with the anodic and cathodic peak current of 12.23 and 9.02 μA, respectively. After the modification of GCE by MnO_2_NFs or NrGO, the redox peak currents increased by 2-fold approximately. As expected, a well-defined and sharp redox peak was observed at the MnO_2_NFs/NrGO/GCE, with the highest anodic and cathodic peak currents (*i*_pa_ = 92.41 μA, *i*_pc_ = 87.89 μA). It indicates that MnO_2_NFs/NrGO significantly improved electrochemical performances. It is well-known that the effective electroactive area is a critical factor that directly influences the electrochemical sensing performances. The effective electroactive areas of different electrodes were also calculated, using the *Randles*–*Sevcik* equation as follows [16,17,20]:

*i*_pc_ = (2.69 × 10^5^) *n*^3/2^ D^1/2^*v*^1/2^*AC* (1)

where *i*_pc_ represents the cathodic peak current (A), *n* represents the electron transfer number, D represents the diffusion coefficient of K_3_[Fe(CN)_6_] (7.6 × 10^−6^ cm^2^ s^−1^ [39]), *v* denotes the scanning rate (V s^−1^), A denotes the effective electroactive area (cm^2^), and *C* denotes the K_3_[Fe(CN)_6_] concentration (mol cm^−3^). The effective electroactive areas were estimated to be 0.0770, 0.3183, 0.3958, and 0.7496 cm^2^ for the bare GCE, MnO_2_NFs/GCE, NrGO/GCE, and MnO_2_NFs/NrGO/GCE, respectively. The effective electroactive area of MnO_2_NFs/NrGO/GCE is about 9-fold higher than that of the bare GCE approximately. The results suggest that the MnO_2_NFs/NrGO nanocomposites significantly enlarged the effective electroactive surface area, which promoted the accumulation of target analysts and thus increased the response electrochemical signals. 

EIS has intensively been used to investigate interfacial properties of various electrochemical sensors [16,17,40,41,42]. Nyquist plots for various electrodes are plotted in Figure 3B. Obviously, Nyquist plots comprise of the semicircular at higher frequencies relating to the electron transfer-limited process, and linear portions at lower frequencies corresponding to the diffusion-controlled process. The semicircular diameter represents the charge transfer resistance (*R*_ct_). The *R*_ct_ values for the bare GCE, MnO_2_NFs/GCE, NrGO/GCE, and MnO_2_NFs/NrGO/GCE are 1950, 2551, 72.47, and 25.28 ohm, respectively. After modification with MnO_2_NFs, the *R*_ct_ value increased by 601 ohm because of the poor electro-conductivity of MnO_2_. When GCE was modified with NrGO, the *R*_ct_ significantly decreased to 74.25 ohm, which can attribute to the good electro-conductivity and high-specific surface area of NrGO [35,36]. As expected, the lowest *R*_ct_ value was obtained at MnO_2_NFs/NrGO/GCE, probably due to the existence of abundant electrocatalytic active sites that can greatly accelerate the redox reaction of [Fe(CN)_6_]^3−/4−^. The results demonstrate that the MnO_2_NFs/NrGO can effectively decrease the *R*_ct_. 

### 3.3. Voltammetric Responses of DA and UA at Various Electrodes

**Figure 4:**
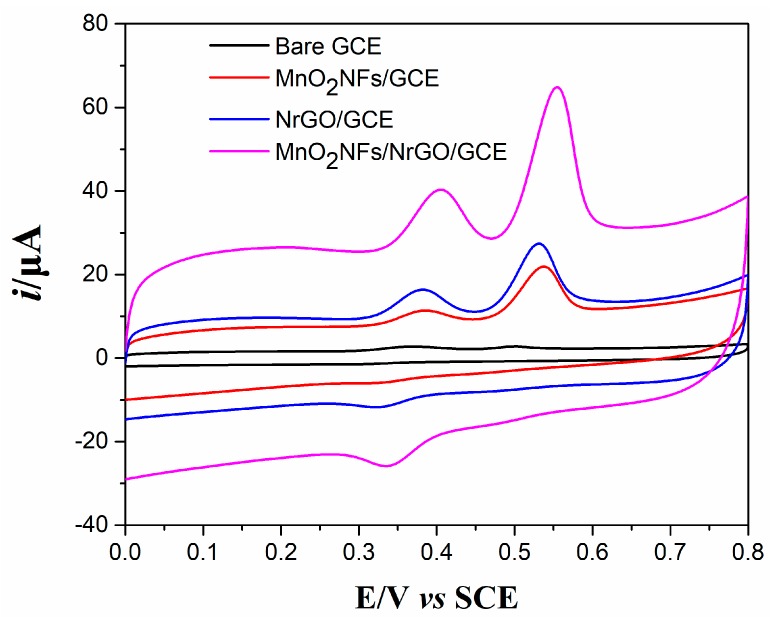
CVs of 10 μM Dopamine (DA) and uric acid (UA) mixture solution (1:1) on different electrodes.

CV responses of 10 μM DA and UA (1:1) were measured at different electrodes in 0.1 M PBS (pH 3.93) (Figure 4). When the potentials were scanned from 0 to 0.8 V, two anodic peaks were observed at all electrodes, which are closely related to the oxidation of DA and UA. However, only one peak belonging to the reduction of DA occurred at reverse scanning. These phenomena indicate that the electrooxidation of UA is totally irreversible. On the bare GCE, two very weak anodic peaks appeared (*i*_pa_(DA) = 1.126 μA, *i*_pa_(UA) = 0.385 μA), demonstrating sluggish kinetics for the electrooxidation of DA and UA. After modification of the GCE by MnO_2_NFs, the *i*_pa_(DA) increased a little (*i*_pa_(DA) = 3.766 μA) while the i_pa_(UA) was enhanced significantly (i_pa_(UA) = 12.73 μA), showing MnO_2_NFs have good electrocatalytic toward the oxidation of UA because of the presence of Mn^4+^/Mn^3+^ as an electron mediator. Moreover, the high-specific surface area also contributed to the obvious enhancement on the *i*_pa_(UA). When GCE was modified with NrGO, the *i*_pa_(DA) and *i*_pa_(UA) increased to 7.029 μA and 16.43 μA respectively, suggesting superb electrocatalytic activity toward the oxidation of DA and UA. The superb electrocatalytic activity of NrGO can explain the following facts. Nitrogen atoms in NrGO sheets may interact with target biomolecules via hydrogen bond, which can activate the amine and hydroxy groups and expedite the charge transfer process. Meanwhile, the π–π interactions between NrGO and target biomolecules can also facilitate the charge transfer process [43]. Two sharp anodic peaks occurred at MnO_2_NFs/NrGO/GCE, and the anodic peak currents enhanced remarkably (*i*_pa_(DA) = 14.8 μA, *i*_pa_(UA) = 36.3 μA). The synergistic effect between MnO_2_NFs and NrGO sheets was mainly responsible for the enhanced response peak currents. Specially, MnO_2_NFs had higher catalytic activity toward the oxidation of DA and UA when the electrical conductivity was improved by coupling with NrGO sheets. Meanwhile, the hydrogen bond and π–π interactions between NrGO sheets and target biomolecules can also facilitate the charge transfer process. It is worth noting the biggest peak potential separation (about 150 mV) at MnO_2_NFs/NrGO/GCE, rendering this composite more selective for the simultaneous detection of UA and DA. Besides, the largest background current was also obtained at the MnO_2_NFs/NrGO nanocomposites, due to the high-specific capacitance of the MnO_2_NFs/NrGO nanocomposites [44].

### 3.4. Optimization of Voltammetrical Parameters

#### 3.4.1. Effect of pH

**Figure 5:**
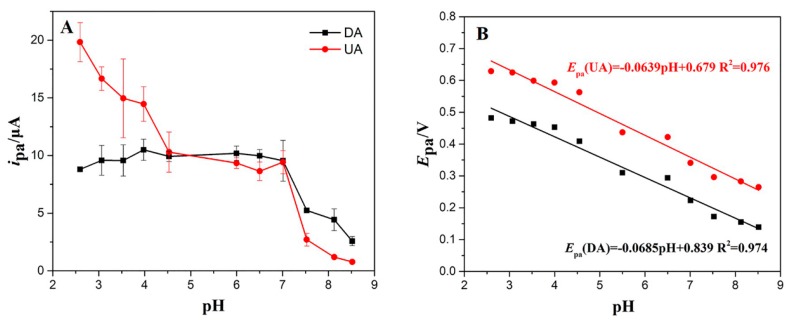
(**A**) Dependence of pH on the anodic peak currents of DA and UA. (**B**) Linear plots of anodic peak potentials of DA and UA against pH (*n* = 3).

**Scheme 1:**
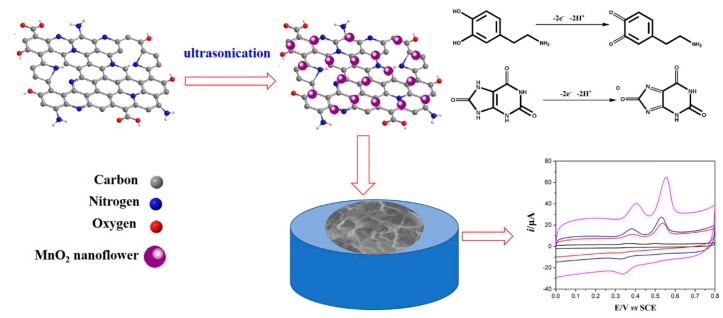
Scheme diagram of the electrochemical oxidation of DA and UA at the MnO_2_NFs/NrGO/GCE.

As known to all, the voltammetric responses of DA and UA highly depend on the solution pH. Therefore, it’s worthwhile to optimize pH. The dependences of pH on the anodic peak currents of DA and UA are shown in Figure 5A. In the pH range of 2.58 to 3.93, the *i*_pa_(DA) gradually increased with the increase of pH, then decreased slowly as the pH rose to 7.01, and suddenly decreased when the pH exceeded 7.01. Obviously, the maximal *i*_pa_(DA) was achieved at pH 3.93. As for UA, the *i*_pa_(UA) show a downward trend, with pH varying from 2.58 to 8.52. To ensure the highest possible anodic peak current for DA and UA, pH 3.93 was selected for the following experiments. Moreover, the anodic peak potentials of DA and UA linearly decreased as pH was rising (Figure 5B). The linear relationships of *E*_pa_ versus pH can be expressed as *E*_pa_(DA) = −0.0685 pH + 0.839 (R^2^ = 0.974) and *E*_pa_(UA) = −0.0639 pH + 0.679 (R^2^ = 0.976), respectively. Their slopes (68.5 pH/mV and 63.9 pH/mV) are close to 59 mV/pH, demonstrating the equal numbers of electron (e^−^) and protons (H^+^) involved in their electrooxidation processes [16]. As reported, the oxidation of DA and UA are two electron transfer processes [45]. Hence, the electrooxidation of DA and UA involves two electrons (2e^−^) and two protons (2H^+^). The electrochemical oxidation process of DA and UA at the MnO_2_NFs/NrGO/GCE are illustrated in Scheme 1.

#### 3.4.2. Effect of Scanning Rate

**Figure 6:**
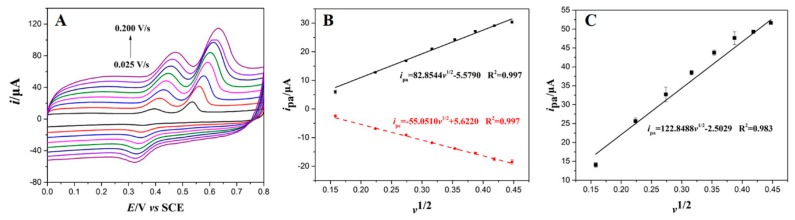
(**A**) CVs of 10 μM DA and UA (1:1) at different scanning rates. (**B**) Linear relationship between the redox peak currents of DA and square root of scanning rates (*n* = 3); (**C**) Linear relationship between the anodic peak currents of UA and square root of scanning rates (*n* = 3).

In order to give a deep insight into the oxidation of DA and UA, CVs of 10 μM DA and UA were performed at various scanning rates (Figure 6A). As the scanning rates increased, their anodic peaks shifted positively while the cathodic peaks shifted in the reverse direction. Furthermore, their response peak currents increased with the potentials scanning speeding up. It is noteworthy that the background currents also enhanced synchronously. To pursue high-signal to noise (S/N), 0.1 Vs^−1^ was recommended as the optimal scanning rate. As shown in Figure 6B, the redox peak currents of DA were proportional to the square root of the scanning rate (*v*^1/2^), suggesting the electrooxidation of DA is a diffusion-controlled process. There was also a good relationship between the anodic peak currents of UA and the square root of the scanning rate (Figure 6C), indicating a diffusion-limited electrode process for UA oxidation. 

#### 3.4.3. Influence of Accumulation Parameters

**Figure 7:**
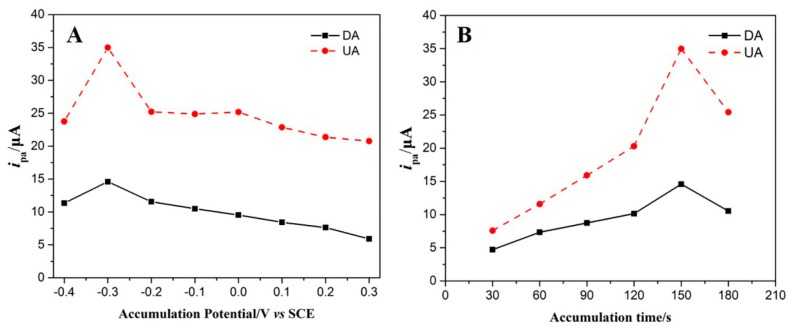
Effect of accumulation potential (**A**) and time (**B**) on the anodic peak currents of DA and UA.

Accumulation can effectively boost the response peak currents of target species, so the influence of accumulation potential and time were also investigated. The anodic peak currents of DA and UA sharply increased with the accumulation potentials shifting from −0.4 V to −0.3 V, then they gradually decreased with a further increase of the accumulation potential (Figure 7A). The highest anodic peak currents of DA and UA were achieved at −0.3 V, so −0.3 V was chosen as the optimal accumulation potential. As presented in Figure 7B, their anodic peak currents gradually enhanced during the first 150 s, then decreased with the prolonging of the accumulation time. Therefore, accumulation was performed at −0.3 V for 150 s in the following experiments.

### 3.5. Individual and Simultaneous Determination of DA and UA

**Figure 8:**
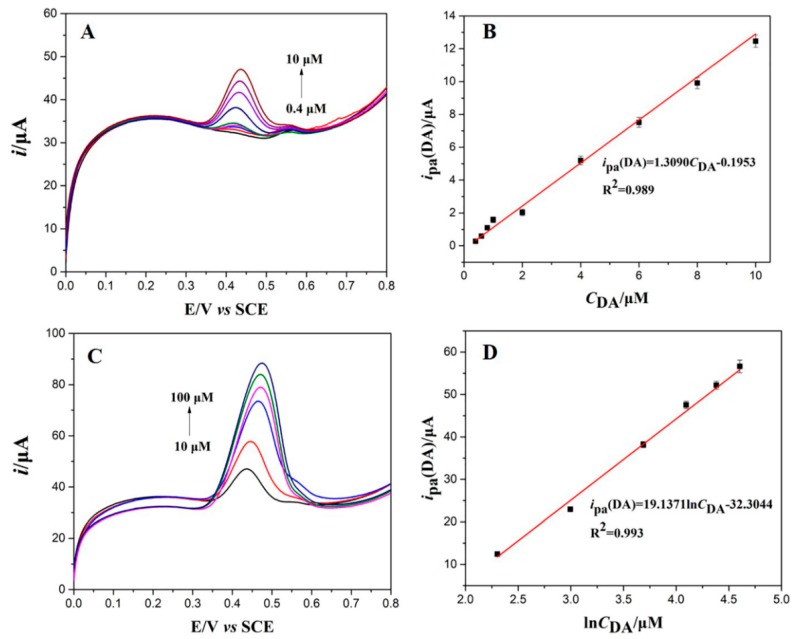
Linear scanning voltammetry (LSVs) at the MnO_2_NFs/NrGO/GCE in 0.1 M PBS (pH 3.93) containing 1 μM UA and various DA concentrations from 0.4 μM to 10 μM (**A**) and from 10 μM to 100 μM (**C**); Linear plots of the *i*_pa_(DA) versus the DA concentrations varying from 0.4 μM to 10 μM (**B**) and from 10 μM to 100 μM (**D**).

**Figure 9:**
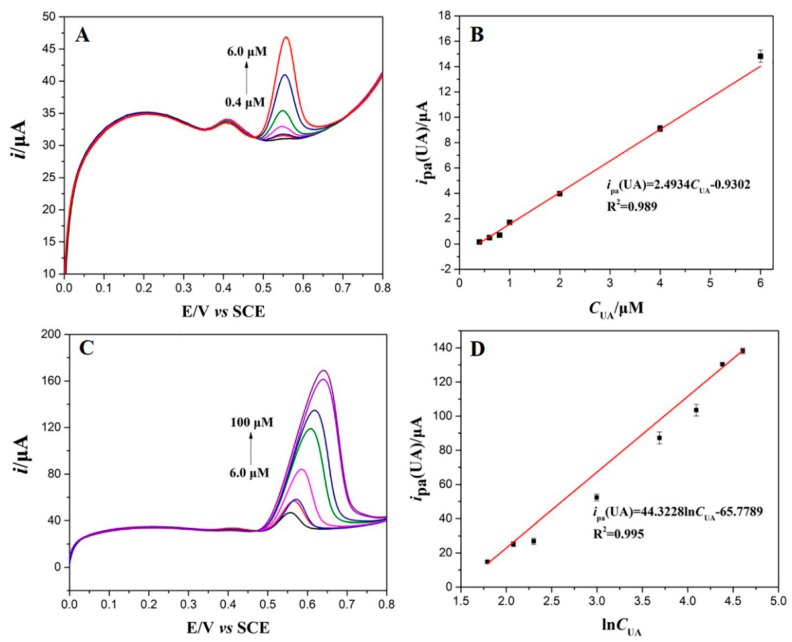
(**A**) LSVs at the MnO_2_NFs/NrGO/GCE in 0.1 M phosphate buffered solution (PBS) (pH 3.93) containing 1 μM DA and various UA concentrations from 0.4 μM to 6.0 μM (**A**) and from 6.0 μM to 100 μM (**C**) (*n* = 3); Linear plots of the *i*_pa_(UA) versus the UA concentrations varying from 0.4 μM to 6.0 μM (**B**) and from 6.0 μM to 100 μM (**D**) (*n* = 3).

For individual detection of DA and UA at the MnO_2_NFs/NrGO/GCE, LSVs were measured in the potential range of 0–0.8 V in 0.1 M PBS (pH 3.93). In this case, only the concentrations of the target substance were varied, while the concentrations of the other substance were kept unchanged. As illustrated in Figure 8A,B, there was a good linear relationship between the *i*_pa_(DA) and DA concentrations ranging from 0.4 μM to 10 μM. The linear equation is *i*_pa_(DA) = 1.3090*C*_DA_ − 0.1953 (R^2^ = 0.989). However, the *i*_pa_(DA) were positively proportional to the Napierian logarithm of DA concentrations (ln*C*_DA_) in the concentration range of 10 μM–100 μM (Figure 8C,D). The linear regression equation can be expressed as *i*_pa_(DA) = 19.1371ln*C*_DA_ − 32.3044 (R^2^ = 0.993). The LOD was estimated as 0.054 μM. Regarding the individual determination of UA, the *i*_pa_(UA) were well-linear to the working concentrations, varying from 0.4 μM to 6.0 μM (Figure 9A,B), with the linear equation of *i*_pa_(UA) = 2.4934*C*_DA_ − 0.9302 (R^2^ = 0.989). At the higher concentration region (6.0 μM to 100 μM), the *i*_pa_(UA) were positively correlated to the Napierian logarithm of UA concentrations (ln*C*_UA_). The corresponding linear equation is *i*_pa_(UA) = 44.3228ln*C*_UA_ − 65.7789 (R^2^ = 0.995). The LOD is 0.062 μM for the individual determination of UA. It is noteworthy that the addition of the target biomolecule does not have a notable interference on the electrochemical response signals of the other biomolecule. The results firmly imply that DA and UA can be sensitively and selectively detected at MnO_2_NFs/NrGO/GCE in the mixture of DA and UA. 

**Figure 10:**
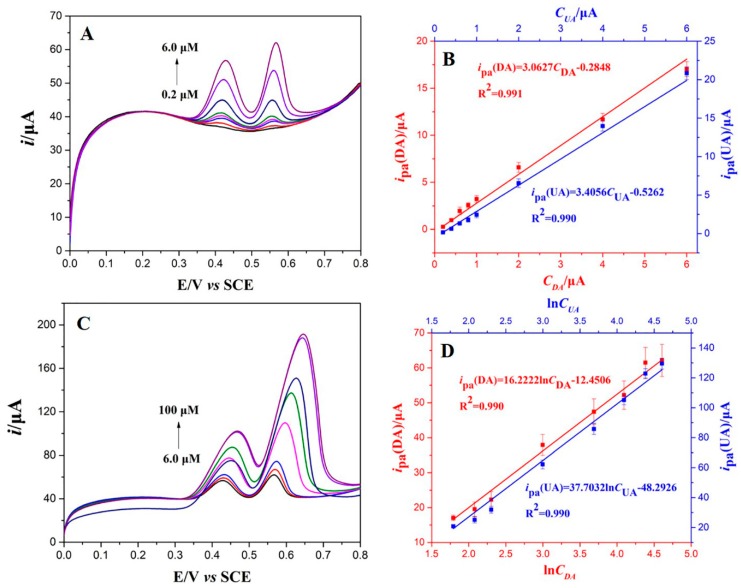
(**A**) LSVs on the MnO_2_NFs/NrGO/GCE in 0.1 M PBS (pH 3.93) containing various concentrations of DA and UA ranging from 0.2 μM to 6.0 μM (**A**) and from 6.0 μM to 100 μM (**C**); (**B**) Plots of the anodic peak currents as the function of DA and UA concentrations in the range of 0.2 μM –6.0 μM (*n* = 3); (**D**) Plots of the anodic peak currents as the function of DA and UA concentrations in the range of 6.0 μM–100 μM (*n* = 3).

The remarkable electrocatalytic activity of MnO_2_NFs/NrGO enables simultaneous detection of DA and UA using the LSV method (Figure 10). Two well-separated anodic peaks belonging to the electrooxidation of DA and UA were observed on LSV curves using MnO_2_NFs/NrGO/GCE. Furthermore, LSV responses were resolved into two peaks at 0.420 V and 0.554 V, respectively. The results demonstrate an excellent discriminability from the two biomolecules in mixture solutions. At the lower concentration region (0.02 μM–6.0 μM), the *i*_pa_(DA) and *i*_pa_(UA) enhanced linearly with their concentrations increasing (Figure 10A, B). The linear plots can be expressed as *i*_pa_(DA) = 3.0627*C*_DA_ − 0.2848 (R^2^ = 0.991), and *i*_pa_(UA) = 3.0627*C*_UA_ − 0.2848 (R^2^ = 0.990), respectively. However, the *i*_pa_(DA) and *i*_pa_(UA) are positively proportional to the Napierian logarithm of the DA and UA concentrations (ln*C*_DA_ and ln*C*_UA_) at the higher concentration region from 6.0 μM to 100 μM (Figure 10C, D). The corresponding linear regression equations are *i*_pa_(DA) = 16.2222ln*C*_DA_ − 12.4506 and *i*_pa_(UA) = 37.7032ln*C*_UA_ − 48.2926, respectively. The correlation coefficient is 0.990 for both DA and UA. The LODs are calculated to be 0.036 μM and 0.029 μM for DA and UA, respectively. All of the results indicate that the proposed MnO_2_NFs/NrGO/GCE featured wider linear detection ranges and a lower LOD for the electrochemical oxidation of DA and UA. The analytical performances were compared to those in previous works (Table 1). Obviously, the sensing parameters of the proposed sensor are comparable to, or even better than, previously reported modified electrodes [7,46,47,48,49,50,51,52,53,54]. 

### 3.6. Selectivity, Repeatability, and Reproducbility Assay

Before real sample detection, the selectivity and repeatability, as well as reproducibility were also evaluated. To assess the anti-interfering ability of the proposed MnO_2_NFs/NrGO/GCE, the LSV responses of the DA and UA in the coexistence of common interfering substances (i.e., ascorbic acid, alanine, citric acid, glutamic acid, cysteine, and lysine) were compared. The relative errors (less than 5%) were accepted even in the presence of 100-fold the above interfering species, demonstrating good selectivity (Figure S1). It is noted that the 100-fold AA have no obvious interfering because of the well-separated peak potential between AA and DA (∆*E*_p_ = 0.260 V). To check the repeatability, eight successive determinations of 1 μM DA and UA (1:1) were also performed. The relative standard deviation (RSD) for DA and UA were 6.35% and 5.32%, respectively, indicating good repeatability. To examine electrode reproducibility, the anodic peak currents of 1 μM DA and UA were recorded at five MnO_2_NFs/NrGO/GCEs, which were prepared by similar procedure. The RSD of the anodic peaks were 6.02% and 4.70% for DA and UA respectively, showing that the electrode preparation have excellent reproducibility.

### 3.7. Determination of DA and UA in Human Serum Samples

To validate practicability, the concentrations of DA and UA in human serum samples were also detected on MnO_2_NFs/NrGO/GCE. The determination results were calculated from the calibration curves (Table 2). To further validate the precision of the proposed sensor, a series of known concentration solutions of DA and UA were spiked with the serum samples to figure out the recovery. The recoveries are 96.2–105.6% and 96.2–104.9% for DA and UA respectively, verifying that biological matrixes, like human serum, do not influence the simultaneous detection of DA and UA.

## 4. Conclusions

In summary, this paper reported the facile synthesis of MnO_2_NFs/NrGO nanocomposites and their application on the simultaneous determination of DA and UA. The electrochemical measurements indicated that the MnO_2_NFs/NrGO composites possess a large, effective electroactive area and low-charge transfer resistance. MnO_2_NFs/NrGO/GCE showed superb catalytic capacity toward the electrooxidation of DA and UA, attributing to the synergistic effect from MnO_2_NFs and NrGO sheets. The anodic peak currents of DA and UA increased linearly with their concentrations varying from 0.2 μM to 6.0 μM. However, their anodic peak currents were highly correlated to the Napierian logarithm of their concentrations, ranging from 6.0 μM to 100 μM. The LODs were 0.036 μM and 0.029 μM for DA and UA, respectively. Furthermore, the proposed sensor successfully realized DA and UA detection in human serum samples with satisfactory recovery. Combining with prominent advantages such as facile preparation, high sensitivity, good selectivity, repeatability, and reproducibility, the proposed MnO_2_NFs/NrGO nanocomposites have become the most competitive candidates for the simultaneous determination of DA and UA in various real samples.

## Figures and Tables

**Table 1:** Comparison sensing performance between previous reports and the proposed MnO_2_NFs/NrGO/GCE for the simultaneous detection of DA and UA.

Electrodes	Methods	Detection Range (μM)		LOD (μM)		Ref.
		DA	UA	DA	UA	
Au/Cu_2_O/rGO/GCE	^a^ DPV	10–90	100–900	3.9	6.5	[7]
NrGO/GCE	DPV	0.5–170	0.1–20	0.25	0.045	[46]
Pd/RGO/GCE	DPV	0.45–71	6–469.5	0.18	1.6	[47]
Pt/RGO/GCE	DPV	10–170	10–130	0.25	0.45	[48]
ZnO/SPCE	DPV	0.1–374	0.1–169	0.004	0.00849	[49]
PtNi@MoS_2_/GCE	DPV	0.5–150	0.5–600	0.1	0.1	[50]
Au–Pt/GO–ERGO	DPV	0.0682–49,800	0.125–82,800	0.0207	0.0407	[51]
HFP/GCE	DPV	1–200	20–400	0.016	0.218	[52]
MoS_2_/GCE	DPV	1–900	1–60	0.15	0.06	[53]
ZnO/PANI/rGO/GCE	DPV	0.1–90	0.5–90	0.017	0.12	[54]
MoS_2_/rGO/GCE	DPV	5–545	25–2745	0.05	0.46	[55]
NCNF/GCE	DPV	1–10; 10–200	5–200	0.5	1	[56]
PTPCNs/GCE	DPV	1–100	5–200	0.078	0.17	[57]
MnO_2_NFs/NrGO/GCE	LSV	0.2–6.0; 6.0–100	0.2–6.0; 6.0–100	0.036	0.029	This work

^a^ DPV: differential pulse voltammetry.

**Table 2:** Detection results of DA and UA in human serum samples using MnO_2_NFs/NrGO/GCE (*n* = 3).

^a^ Samples		Detected (μM)	Added (μM)	Found (μM)	RSD (%)	Recovery (%)
**Serum**	DA	^b^ ND	20	18.98	4.25	94.9
			40	38.48	3.76	96.2
	UA	21.8	20	42.92	2.98	105.6
			40	63.74	2.45	104.9

^a^ The human serum samples were detected at 10-fold dilution. ^b^ Not detected.

## Supplementary Materials

The following are available online at https://www.mdpi.com/2079-4991/9/6/847/s1, Figure S1: The anodic peak currents of 1 μM DA and UA in the presence of 100-fold alanine (AL), glutamic acid (GA), ascorbic acid (AA), lysine (LY) and citric acid (CA).
