# Supplementary material for: Facile Synthesis of MnO2 Nanoflowers/N-Doped Reduced Graphene Oxide Composite and Its Application for Simultaneous Determination of Dopamine and Uric Acid
Source: Nanomaterials (Basel). 2019 Jun 2;9(6):847. doi: 10.3390/nano9060847 (PMC6631201; doi:10.3390/nano9060847)
Supplement: Supplementary file 1 [file nanomaterials-09-00847-s001.pdf]

Supporting Information

Facile Synthesis of MnO_2 Nanoflowers/N-Doped Reduced Graphene Oxide Composite and Its Application for Simultaneous Determination of Dopamine and Uric Acid

Xuan Wan [1,†], Shihui Yang [1,†], Zhaotian Cai [1], Quanguo He [1], Yabing Ye [1], Yonghui Xia [2], Guangli Li [1,*], and Jun Liu [1,*]

[1] College of Life Sciences and Chemistry, Hunan University of Technology, Zhuzhou 412007, China; wanxuan1111@163.com (X.W.); yangshihui0522@163.com (S.Y.); caizhaotian1998@163.com (Z.C.); hequanguo@126.com (Q.H.); yyb980501@163.com (Y.Y.)

[2] Zhuzhou Institute for Food and Drug Control, Zhuzhou 412000, China; Sunnyxia0710@163.com

* Correspondence: guangli010@hut.edu.cn (G.L.); junliu@hut.edu.cn (J.L.); Tel.: +86-0731-2218-3382 (G.L. & J.L.)

† These authors contributed equally to this work.

Received: 13 May 2019; Accepted: 28 May 2019; Published: date

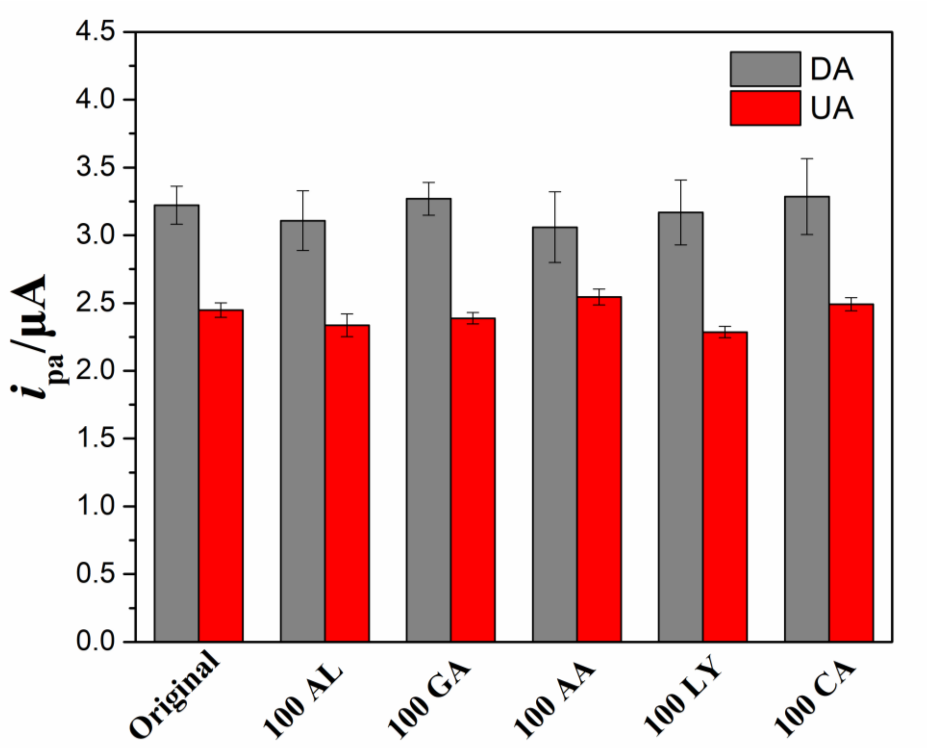

Figure S1. The anodic peak currents of 1 μM DA and UA in the presence of 100-fold alanine (AL), glutamic acid (GA), ascorbic acid (AA), lysine (LY) and citric acid (CA).
